# Editorial: Discovery and Total Synthesis of Bio-functional Natural Products From Traditional Medicinal Plants

**DOI:** 10.3389/fchem.2020.00650

**Published:** 2020-08-21

**Authors:** Toshio Morikawa, Satoru Tamura, Tao Wang

**Affiliations:** ^1^Pharmaceutical Research and Technology Institute, Kindai University, Osaka, Japan; ^2^School of Pharmacy, Iwate Medical University, Iwate, Japan; ^3^Institute of TCM, Tianjin University of Traditional Chinese Medicine, Tianjin, China

**Keywords:** natural products chemistry, phytochemistry, total synthesis, bioorganic chemistry, isolation, structural determination

The term “natural product” refers to any naturally occurring substance, but it generally refers to a secondary metabolite (Morikawa, 2019; Sicker et al., 2019). Secondary metabolites, which are isolated from plants, animals, and microorganisms, are classified as polyketides, isoprenoids, steroids, aromatics, and alkaloids. Research into the discovery and synthesis of novel bio-functional natural products is a challenging, expensive, and time-consuming process (Pagare et al., 2015; Seca and Pinto, 2019). However, research on natural products stimulates the development of novel separation techniques, spectroscopic approaches to structure elucidation, and synthetic methodologies. The chemical diversity and variety of bio-functional properties of natural products thus attracts attention from chemists, biochemists, and biologists (Morikawa, 2018a,b). The Research Topic on “Discovery and Total Synthesis of Bio-Functional Natural Products from Traditional Medicinal Plants” is intended to promote bio-functional natural products as candidates and/or leads for pharmaceuticals, nutraceuticals, dietary supplements, cosmetics, and food additives. The field of this Research Topic includes natural products chemistry, phytochemistry, pharmacognosy, organic chemistry, food chemistry, bioorganic chemistry, chemical biology, molecular pharmacology, molecular nutritional sciences, and related fields of bio-functional natural products.

The review by Grynkiewicz and Demchuk discussed new perspectives for fisetin, a naturally occurring flavonol, which has distinct antioxidant properties along with a plethora of other plant polyphenols. In particular, they described the potential applications and demand for fisetin in healthcare, methods for its preparation, and its suitability for pharmaceutical use. Wang, Song et al. reviewed the phytochemical, structural modification, and relevant bioactivities, such as anticancer, lipid-regulating, anti-inflammatory, antibacterial, antiviral, and diuretic activities of triterpenoids, especially those obtained from *Alisma* species and their derivatives. Harada et al. summarized the chemistry and neurotrophic activities of (–)-talaumidin and its derivatives. They achieved the first enantioselective total synthesis of (–)-talaumidin *via* a flexible and reliable synthetic pathway involving an Evans asymmetric aldol reaction, as well as stereo-controlled hydroboration and Friedel-Crafts arylation, to construct the four contiguous chiral centers on the tetrahydrofuran ring. To investigate the structure-activity relationships, a systematic synthesis of all diastereomers and syntheses of several related derivatives was reported along with an evaluation of neurite outgrowth promotion and neuroprotection in primary cultured rat cortical neurons and in nerve growth factor-differentiated PC12 cells. Candidates including (–)-talaumidin for the regeneration of mouse optic nerves *in vivo* were discovered. Nakamura et al. developed a practical and reproducible method for total synthesis of hydroxy-α-sanshool, α-sanshool, and spilanthol, which is a characteristic polyunsaturated fatty acid amide obtained from *Zanthoxylum* species. Notably, a highly selective Wittig reaction using a newly synthesized phosphonium salt with low deliquescence and long-term stability created the desired *Z*-form polyenes. This improved methodology was shown to be applicable to the efficient synthesis of other sanshool derivatives. Regarding the structure determination of novel naturally occurring compounds from traditional medicinal plants, four picrotoxane-type sesquiterpenes, dendroterpene A–D, were isolated from the stems of *Dendrobium nobile* (Wang, Chen et al.). Six highly oxidized lanostane- and cycloartane-type triterpenes, xuetongalactones A–F, were obtained from the stems of *Kadsura heteroclita* (Shehla et al.). Six new limonoids, dictamlinonol A, dictamlimonoside B, and dictamlimonols C–F, were isolated from the root bark of *Dictamnus dasycarpus* (*Cortex Dictamni*) (Chen et al.). Three new geranylated coumarins, kayeassamin I and mammeasins E and F, were obtained from the flowers of *Mammea siamensis* (Morikawa et al.). Five new cyclic organosulfer compounds, foliogarlic disulfanes A_1_-A_3_ and foliogarlic trisulfanes A_1_ and A_2_, were isolated from the leaves of *Allium sativa* (Fukaya et al.). Their structures, including the stereochemistry, were elucidated by NMR, MS, X-ray diffraction, and electronic circular dichroism spectroscopic analyses. Various bio-functional activities including; α-glucosidase inhibitory (Wang, Chen et al.), cytotoxicic (Wang, Chen et al.; Shehla et al.), anti-inflammatory (Shehla et al.; Chen et al.), and 5α-reductase inhibitory activities (Morikawa et al.) were also reported. A limonoid fraxinellon was a noteworthy compound obtained as a main constituent of *Cortex Dictamni* in a yield of ~0.15% with remarkable anti-inflammatory activity. Fraxinellon inhibited lipopolysaccharide (LPS)-induced nitric oxide production and reduced the LPS-induced expression of inducible nitric oxide synthase and cyclooxigenase-2 at the mRNA and protein levels in a dose-dependent manner by regulating the nuclear factor kappa-light-chain-enhancer of activated B cells in RAW 264.7 macrophage-like cells (Chen et al.). In addition, a geranylated coumarin surangin C obtained from *M. siamemsis* flowers exhibited 5α-reductase inhibitory activity (IC_50_ = 5.9 μM). Although the intensity of the 5α-reductase inhibitory activity of these coumarins is moderate compared to the positive control with a steroid skeleton finasteride (IC_50_ = 0.12 μM), there are few reports of 5α-reductase inhibitors with non-steroidal skeletons. These active coumarins may therefore be useful candidates for seed compounds of new non-steroidal 5α-reductase inhibitors (Morikawa et al.).

Exploratory research using naturally occurring products with diverse chemical and bio-functional properties remains a promising tool for discovering new bio-functional principles. The isolation and structural elucidation of the constituents from a wide variety of medicinal resources, including traditional medicinal plants, as well as synthetic research and biological evaluation are therefore being performed. Fortunately, in this Research Topic, researchers who are active in this field have reported on recent promising research. We hope that the articles collected within this Research Topic will help inspire readers to embrace future opportunities in the field.

## Author Contributions

All authors listed have made a substantial, direct and intellectual contribution to the work, and approved it for publication.

## Conflict of Interest

The authors declare that the research was conducted in the absence of any commercial or financial relationships that could be construed as a potential conflict of interest.
